# Novel Organogels from *Mauritia flexuosa* L.f and *Caryodendron orinocense* Karst.: A Topical Alternative

**DOI:** 10.3390/pharmaceutics15122681

**Published:** 2023-11-27

**Authors:** Luis Eduardo Mosquera Narvaez, Marcela P. Carrillo, Juliana E. C. Cardona-Jaramillo, Bibiana Margarita Vallejo, Lindalva Maria de Meneses Costa Ferreira, José Otávio Carréra Silva-Júnior, Roseane Maria Ribeiro-Costa

**Affiliations:** 1 Institute of Health Sciences, Federal University of Pará, Belém 66075-110, Brazil; luis.narvaez@ics.ufpa.br (L.E.M.N.); lindalva.costa.ferreira@ics.ufpa.br (L.M.d.M.C.F.); carrera@ufpa.br (J.O.C.S.-J.); 2 Sinchi Amazon Research Institute, Bogotá 110311, Colombia; mcarrillo@sinchi.org.co (M.P.C.); jcardona@sinchi.org.co (J.E.C.C.-J.); 3 Health Sciences Institute, National University of Colombia, Bogotá 111321, Colombia; bmvallejod@unal.edu.co

**Keywords:** *Mauritia flexuosa*, *Caryodendron orinocense*, cetostearyl alcohol, organogel, topical application, natural ingredients

## Abstract

Organogels have importance for topical applications because they can be used to deliver drugs in a controlled and prolonged fashion. These are materials consisting of a three-dimensional network of organic molecules dispersed in a solvent. Recent studies have demonstrated that the solvent could be replaced by oils from non-conventional biologic sources. There is a diversity of not-explored species in the Amazon that are promising sources of vegetable oils with a promising composition. This study developed an organogel with buriti (*Mauritia flexuosa* L.f) and cacay (*Caryodendron orinocens*e Karst.) oils, using cetostearyl alcohol as an organogelator due to its compatibility, stability, security, affordability, and it is readily available. The oils were characterized, and the organogels were synthesized by studying their crystal evolution and oil-binding capacity. The microstructure was evaluated with polarized light microscopy, fractal dimension, FTIR spectroscopy, XRD, and thermal and rheological analyses. It was found that the critical gelation concentration was higher for cacay oil as it possessed a higher amount of polyunsaturated triacylglycerols. The crystals of the buriti organogel had a smaller lamellar shape, a greater surface area, and physical and thermal stability; although, it presented a slower crystal evolution due to the low number of minor compounds and a greater number of saturated triacylglycerols. The polar fraction of the organogelators as well as triacylglycerol and minor polar compounds are important in forming crystallization nuclei. The study showed that Amazonian oils in crystallization processes form microstructures with differentiating physicochemical properties.

## 1. Introduction

Many skincare-related problems are currently linked to different causes such as free radicals which generate beneficial skin compound oxidation and prematurely aging skin; toxic compounds from the atmosphere which generate rosacea, sensitive skin by potential irritants agents; ultraviolet radiation which generates prematurely aging skin and dry skin [1,2]. Developing new stable formulations with many healthy compounds such as vitamins and essential fatty acids could be a promising option to mitigate these issues. Since Amazonian oils (AO) have been recognized for their abundance of antioxidants, polyunsaturated fatty acids, and vitamins, they have been fostered as a great natural alternative, and organogels (OG) have emerged as a technological option to overcome the current challenges faced in the skincare industry, like formulation stability, ingredient delivery, limited skin penetration, natural and sustainable formulations, texture and sensorial experience, and sensitive skin. Organogels can address those challenges, since they can serve as effective stabilizers, helping to keep the formulation stable and maintain its homogeneity, for example, in the work of Francine and Patricia [3]**,** a cosmetic formulation based on organogel containing sunflower seed oil showed the highest score on sensory evaluation, which firstly suggests that the addition of sunflower seed oil may increase the stability of the organogel system; additionally, organogels can enhance the delivery of ingredients by creating a barrier on the skin that slows down their evaporation or degradation, allowing for better absorption [4]. On the other hand, organogels can be formulated with natural and sustainable ingredients, aligning with the industry’s clean beauty and sustainability trends, providing unique textures and a pleasant sensorial experience. They can make the application of skincare products smoother and can enhance spread ability. Finally, the organogels can be formulated with gentle and hypoallergenic ingredients [5,6], making them suitable for individuals with sensitive skin who may react to traditional formulations.

Organogels are semi-solid systems in which an organic liquid phase is immobilized by a three-dimensional network composed of a polymeric or low molecular weight organogelator (OGG) [7,8,9]. Low molecular weight OGG are typical permeation enhancers; examples include fatty acids, surfactants, alcohols, N-methyl-2-pyrrolidone, urea, sulfoxides, essential oils, terpenes, terpenoids, and glycols [10]. OGG can be classified according to the mechanism of oil binding through the formation of a three-dimensional structure with a fibrillar network or crystalline particles [11,12], formed mainly by aggregation forces between the gelling molecules that include hydrogen bonds, dipole–dipole interactions, π-type interactions, electron transfer, London dispersion forces, hydrophobic effects, and ionic interaction, among others [9,13]. The formation of organogels is performed in two stages: in the first one, the physical organogel is prepared by heating the mixture of the solid and liquid components that forms the organic dispersion solution; then, it proceeds with the cooling of the mixture, which fixes the formation of the gel, known as the second stage. During the latter, the solubility of the structuring agent in the liquid phase decreases, and the OGG–solvent interactions are reduced, which causes the molecules of the structuring agent to remain in the continuous medium in a dispersed form [9,14,15].

The selection of solvents plays a fundamental role in the design and development of new formulations, so over the last decades, a great variety of organic solvents have been studied for their application in the food, pharmaceutical, and cosmetic industries. However, in response to growing consumer interest in natural alternatives, the industry has turned its attention to replacing organic solvents with natural oils. Sourcing natural and sustainable ingredients is a growing concern for the cosmetic industry. Companies are increasingly looking for eco-friendly and ethically sourced ingredients to meet consumer demand for green products. Despite the few studies, these have demonstrated great benefits due to their unique composition and physicochemical and nutraceutical properties. They are widely requested in the cosmetic industry in addition to their traditional uses as humectants, emollients, emulsifiers, and viscosity adjusters [16].

The nutraceutical properties of Amazonian natural oils are not limited to their lipid composition but include the presence of other bioactive compounds that have been called unsaponifiable matter [17]. These molecules are commonly called minor polar constituents (MPC) [12], and they are some fat-soluble vitamins and other antioxidants, which exert a protective action against the evolution of natural degenerative processes that lead to disease and premature aging; for example, tocopherols, carotenoids (provitamin A), phytosterols, and polyphenols are present in various types of natural oils [18]. It has been identified that a low concentration of minor polar compounds in the oil interacts with the organogelator, resulting in stiffer gels, while at higher concentrations of minor polar compounds, a negative impact on gel strength has been observed due to saturation in the interactions with the organogelator [19].

These antioxidants are key to reducing the harmful effects of free radicals generated by oxidative metabolic processes [17]. We highlight buriti oil (BO) and cacay oil (CO) among the most representative Amazonian oils. BO is rich in unsaturated fatty acids [20,21], characterized by high concentrations of nutraceutical compounds, mainly tocopherols (α and β-tocopherol) and carotenoids (α, β and γ-carotene) [21,22,23]. CO has a high content of polyunsaturated fatty acids, distributed in linoleic acid (major component), followed by oleic and linolenic acids. Unsaponifiable compounds such as vitamins E (α, β, γ, and δ-tocopherol), phytosterols (β-sitosterol), and triterpene alcohols are also present [24,25].

The aim of this work is to thoroughly characterize an organogel that utilizes Amazonian oils as solvents for cosmetic application with the end to address the issue of oxidation of beneficial skin compounds, provide skin protection, reduce the incorporation of chemical agents into formulations, and enhance skin nutrition through the presence of nutritional and antioxidant compounds found in Amazonian oil. The results will become fundamental to understanding potential properties, applications, and the process of obtaining the desired structure. The appearance, texture, and consistency of the organogelator versus the thermal stability indicate the future application of such formulations in the controlled release of bioactive compounds through skin barriers. Rheological properties such as elasticity, viscosity, and thixotropy directly influence the application and sensation of the product on the skin identifying benefits such as hydration, softness, and elasticity, among others.

## 2. Materials and Methods

### 2.1. Materials

The *Caryodendron orinocense* oil (CO) and cetostearyl alcohol (CA) were supplied by the Instituto Amazónico de Investigaciones Científicas—Colombia (Sinchi), and the *Mauritia flexuosa* oil (BO) was provided by the Amazon Oil Industry, Ananindeua, Pará- Brazil.

### 2.2. Fatty Acid Profile

Derivatization was conducted according to the protocol by Radice et al. [25] with some modifications. A total of 250 mg of an oil sample were dissolved in 5 mL of methanolic NaOH with 15 mL of preheated derivatization solution (2.0 g of ammonium chlorate, 60 mL of methanol, and 3 mL of sulfuric acid). The solution was heated under reflux for 3 min and then transferred to a separating funnel with 25 mL of hexane and 50 mL of Ultrapurewater. The organic phase was recovered and dried for further analysis in a gas chromatograph coupled to a mass spectrometer (GCMS-QP2010, Shimadzu^®^, Kyoto, Japan) using an RTX5MS column (30 m × 25 mm × 0.25 μm). The injection volume was 1.0 μL, with a split ratio of 50:1, with a flow rate of 1 mL/min using helium as carrier gas. The injector and detector temperature were 300 °C and 240 °C, respectively. The temperature gradient started at 100 °C, at a rate of 5 °C/min until 250 °C, followed by 10 min at 250 °C step. Identification was made using NIST14 library.

### 2.3. Physicochemical Characteristics

The acidity and saponification index were determined using the protocol proposed by the United States Pharmacopeia [26]**;** refractive indices, relative density, and viscosity were determined at room temperature (25 °C) by the Brazil pharmacopeia [27]. The acidity index was carried out by titration with 0.1 N sodium hydroxide solution (KOH), according to the work of Lozano et al. [26]. The saponification index was carried out by titration with 0.5 N potassium hydroxide alcohol solution (KOH) according to the work of Lozano et al. [26]. The refractive index was carried out using a digital refractometer ATAGO PAL-RI at room temperature, and for the calculation of the relative density, a pycnometer of 25 mL was used at room temperature. The viscosity of the oils was measured using a digital viscometer, Lamy Rheology Stainless Steel RM 100 Plus. All analyses were performed in triplicate.

### 2.4. Preparation of Organogelators

Synthesis was performed according to the method of Singh et al. [28] with slight modifications. First, organogels were prepared by dissolving cetostearyl alcohol at concentrations of 1, 3, 5, 7, 9, and 11% (*w*/*w*) in the corresponding oil. Second, the mixtures (oleo-organogelator) were heated at 70 °C under continuous stirring (1000 rpm) until complete melting. Finally, when it reached homogeneity, the system was kept at a constant temperature (60 °C) for 30 min, and then the samples were cooled to room temperature (25 °C) for 24 h and stored at 5 °C. Then samples were kept at room temperature for 24 h before each analysis.

### 2.5. Characterization of Organogel

#### 2.5.1. Evaluation of the Inverted Tube

The formulations prepared at different concentrations of OGG were placed in glass vials and then inverted, after which the presence of adherence to the surface of the glass crystal was visually evaluated, confirming the formation of the organogel.

#### 2.5.2. Microstructure and Appearance

Crystal morphology was determined using a polarized light microscope (Carl Zeiss Microimaging GmbH, Jena, Germany) as described by Alongi et al. [29] and Chen et al. [30]. Images were obtained at 10× and 40× magnification. The microphotographs were analyzed for crystal length (Lc), fractal dimension (D), and lacunarity (A) using Image J Confocal Uniovi 1-51 software (NIH, Bethesda, MA, USA) with the FracLac add-on. The “box-counting” algorithm was used to determine the fractal dimension. For this analysis, it was decided to work with the concentration at which the organogel was formed, which allowed the crystals to be identified more clearly in the visual field of microscopy [31].

#### 2.5.3. Oil Binding Capacity (OBC)

The OBC of organogels was determined with the centrifugation method described by Naeli et al. [32] and Sun et al. [33], with some modifications. Approximately 1.0 g of the sample was transferred to a 1.5 mL Eppendorf tube and placed in a water bath at 70 °C until melted for 30 min to ensure complete removal of its crystalline history; then, the sample was cooled to room temperature and centrifuged in a Hettich EBA 21—Germany-Tuttlingen model centrifuge at 10.000 rpm for 15 min. Excess liquid oil in the tube was removed, and the sample was weighed to measure losses. The process was carried out in triplicate, and the percentage of oil loss was calculated using the following oil released Equation (1)

(1) $$\begin{array}{c} Oil\;released\%=\frac{b-c}{b-a}\\ OBC\%=100-Oil\;released\% \end{array}$$

where *a* is the mass of the centrifuge tube, *b* is the mass of the centrifuge tube and organogel, and *c* is the mass of the centrifuge tube and organogel after centrifugation. A high OBC value means an extraordinary mechanical stability of the organogel, while a low value implies no resistance to stresses and is not considered an organogel.

#### 2.5.4. Fourier Transform Infrared Spectroscopy (FTIR)

FTIR spectra of BO, CO and CA were measured on a Jasco FT-IR-4600 model-Tokyo, Japan at room temperature. Spectra were acquired in a range of 400 and 4000 cm^−1^ at a 2 cm^−1^ resolution. Each sample was measured in duplicate [34].

#### 2.5.5. X-ray Diffraction

X-ray diffraction (XRD) analysis was performed according to the method of Öğütcü et al. [35] with slight modifications. Analysis was performed on an X-ray diffractometer (X’PERT PRO MPD, PANalytical—Almelo, The Netherlands) equipped with a CuKα source (λ = 1.54 Å) operated at 45 kV and 40 mA. Pure organogel was loaded onto a glass slide and then analyzed to record wide-angle X-ray diffraction (WAXS) patterns in the 2θ range from 5° to 70° with a step size of 2°/min.

#### 2.5.6. Crystallization Evolution

The Crystallization evolution was determined according to the method of Sagiri et al. [36] using a UV 1800 spectrophotometer (Shimadzu^®^, Kyoto, Japan) in the 400–700 nm range. The absorbance of the molten organogels was measured for 40 min with an interval of 15 s during the cooling process. The weight of the organogels was kept constant (3 g), and the analysis was performed in triplicate.

#### 2.5.7. Thermal Analysis

Thermal analysis of the organogels was performed in a TA 250 differential scanning calorimeter (DSC) (TA Instruments, Waters LLC, New Castle, DE, USA) to determine the enthalpy of phase transitions, melting, and crystallization points. Approximately 3 mg of sample were placed in hermetically sealed aluminum pans, heated to 80 °C at a 10 °C/min rate, and held for 8 min. The samples were then cooled to 25 °C at a 10 °C/min cooling rate. The nitrogen atmosphere was maintained at 50 mL/min; the data were processed using OriginPro 8.5 2019 software (OriginLab).

#### 2.5.8. Rheological Analysis

Rheological analysis was performed on the OB-CA and OC-CA organogel samples at 25 °C and 37 °C for structural strength evaluation using a Discovery Hybrid Rheometer (DHR) (TA Instruments- New Castle, DE, USA. The selected sensing system used parallel plates with a 1000µm, the conditioning time was 5 min, and the sweep amplitude was from 5 × 10−^3^% to 100% at 10 rad/s. Viscoelastic properties were evaluated with oscillatory measurements; storage modulus (G′), loss (G″), and tan δ were also estimated.

### 2.6. Statistical Analysis

For the statistical analysis, we performed a one-way ANOVA using statgraphics centurion to compare the physicochemical characteristics of the BO and CO with a significance of 5%.

## 3. Results

### 3.1. Analysis of Amazonian Oils

The fatty acid composition of oils is shown in Table 1. The values obtained were within the acceptable range for each type of oil [12]; the highest content of unsaturated fatty acids (UFA) and the lowest content of saturated fatty acids (SFA) was found in CO [25,37] while BO contains the highest amount of monounsaturated fatty acids (MUFA), followed by saturated fatty acids (SFA) [38,39]. The comparison between the physicochemical characteristics of crude buriti oil and cacay oil showed that buriti oil presented higher viscosity and a higher saponification index, while cacay oil presented a higher index of free fatty acids. For the other parameters, we did not find significant differences (*p* ≤ 0.05).

### 3.2. Obtaining Organogels

**Figure 1:**
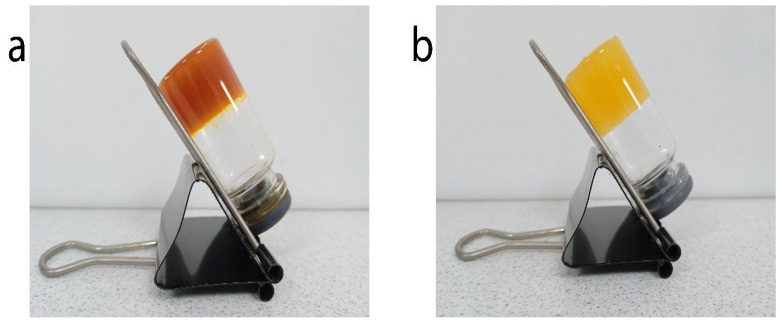
The appearance of organogels: (**a**) Buriti oil with cetostearyl alcohol (9%) and (**b**) Cacay oil with cetostearyl alcohol (11%).

The formation of organogels was confirmed with the inverted tube method, where gel adhesion was observed at the bottom of the vial with a yellow coloration for CO and orange for BO (Figure 1). Formulations made for BO and CO with CA were transparent at 70 °C; as the solution cooled to room temperature (25 °C), it turned opaque. When formulating concentrations below 7% *w*/*w*, phase separation occurred for both CO and BO. It was determined that BO’s critical gelation concentration (CGC) is above 9% *w*/*w*, while CO is above 11%.

### 3.3. Polarized Light Microscopy Analysis

**Figure 2:**
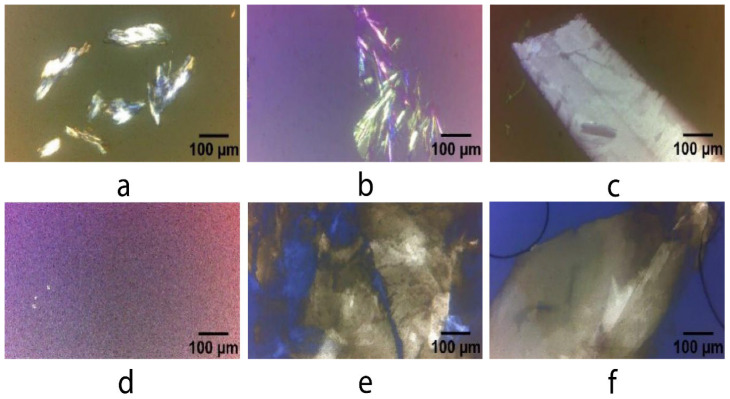
Polarized light micrographs of samples with the first crystal formation: (**a**) CA (5%)-BO; (**b**) CA (7%)-BO; (**c**) CA (9%)-BO; (**d**) CA (7%)-CO; (**e**) CA (9%)-CO; and (**f**) CA (11%)-CO. Scale bar: 100 μm.

The microstructure of the organogels was studied to evaluate the oily medium’s effect on the crystals’ morphology. The morphology and size of crystals formed in the 3D arrangement affect the structural characteristics of the organogels and, subsequently, their application. The crystal formation for each oil occurred at different OGG concentrations. On the one hand, the BO crystal has a morphology that resembles a needle-like conglomerate at the initial stage (5% OGG—Figure 2a), but when the CA concentration reaches a value of 7%, the crystal frequency and size increase (Figure 2b). However, when the OGG reaches 9%, the crystals change their morphology from needle conglomerate to flat crystal (Figure 2c). At concentrations exceeding 9% OGG, the flat geometry remains consistent, accompanied by an observable increase in crystal frequency. This contributes to greater stability within the organogels.

On the other hand, the organogel synthesized with CO generates cross malt at 7% OGG (Figure 2d) and the first polymorphic (allotropic) structure at 9% OGG (Figure 2e) with the formation of large polymorphs with rough and flat structures; at concentrations above 9%, the frequency and size of polymorphs increase (Figure 2f). This behavior may be due to the polyunsaturated nature of the oil, as the non-planar conformation of the fatty acids means that more OGG is required to induce a heterogeneous crystallization nucleus. The nature of the crystal surface is influenced by the hydrophobic forces of these polyunsaturated fatty acids, mainly the London dispersion forces upon cooling [40,41].

**Figure 3:**
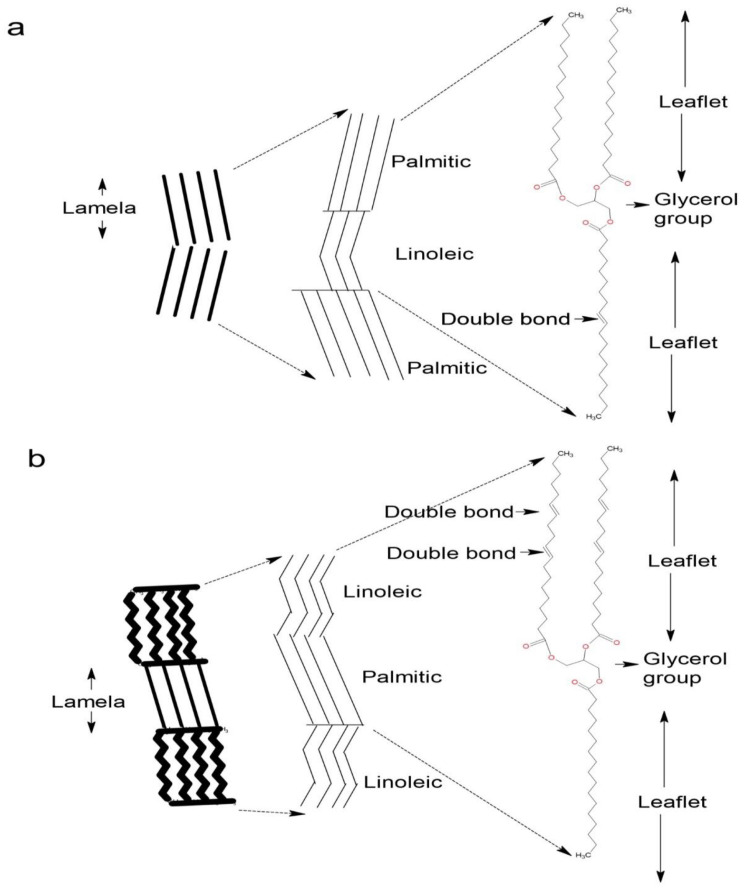
Graphical representation of triacylglycerol structures. (**a**) Triacylglycerol POP (palmitic-oleic-palmitic) structural molecules frequently present in BO. (**b**) LPL (linoleic-palmitic) triacylglycerol structural molecules.

A graphical representation of the generation of the crystal structures for both BO and CO according to their triacylglycerol (TAG) composition can be seen in Figure 3. It has been identified that self-assembly, crystal morphology, and the thermal and viscoelastic properties of organogels depend on a balance between solvent–OGG and OGG–OGG interactions [42].

Table 2 shows the analysis of the organogel images at 10×, where it is possible to clearly see the formation of the crystals for each of the Amazonian oils; the higher the fractal dimension, the more uniformly the mass is distributed, as presented in the BO–CA organogel, which is the network with fewer cavities and more crystals. In addition to the fractal dimension, the lacunarity (A) can also be used to describe certain properties of organogels, as it provides detailed information about the distribution and homogeneity of cavities; lacunarity pertains to both gaps and heterogeneity [39]. In the case of CO–CA organogel, the numerical value of the lacunarity was higher than with BO–CA organogel, indicating that the crystals formed by CO–CA are not widely distributed in the oil space, possibly due to the molecular structure of the fatty acids and mainly due to the presence of a higher amount of polyunsaturated fatty acids that somehow induce the formation of crystals of big size with low crystal frequency. In contrast, saturated fatty acids induce the formation of more crystallization nuclei and, consequently, higher crystal frequency [13,43].

### 3.4. Oil-Binding Capacity (OBC)

**Figure 4:**
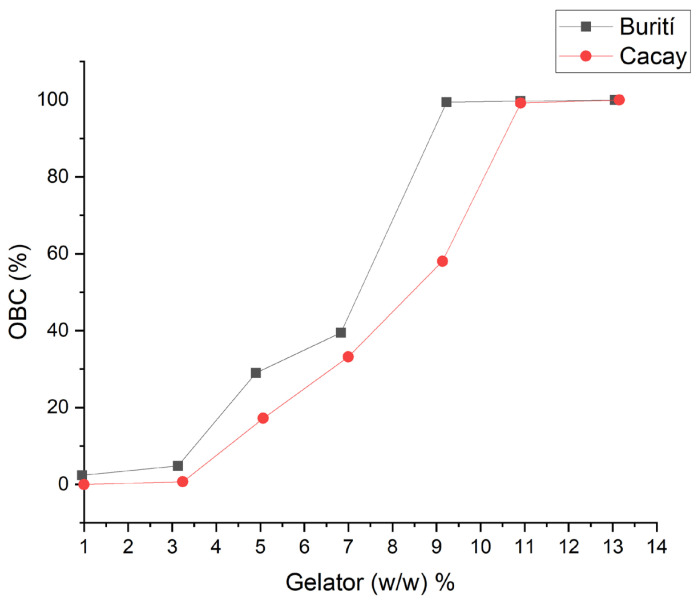
Oil-binding capacity of the organogel of two Amazonian oils: BO–CA and CO–CA.

During the centrifugation process, it was evident that the formed gel exhibited improved visual stability. Testing revealed that concentrations of AC in BO and CO, with values of 9% and 11% have an oil binding capacity of 99.4% and 99.2%, respectively. This enhanced stability can be attributed to the formation of a 3D structure, which effectively reduced precipitation in the centrifugation (Figure 4). The BO–AC organogel suggests the formation of a more consistent 3D structure, as vegetable oils containing TAGs consisting of high melting point saturated fatty acids generate highly viscous organogels making them more resistant [43].

### 3.5. FTIR Analysis

**Figure 5:**
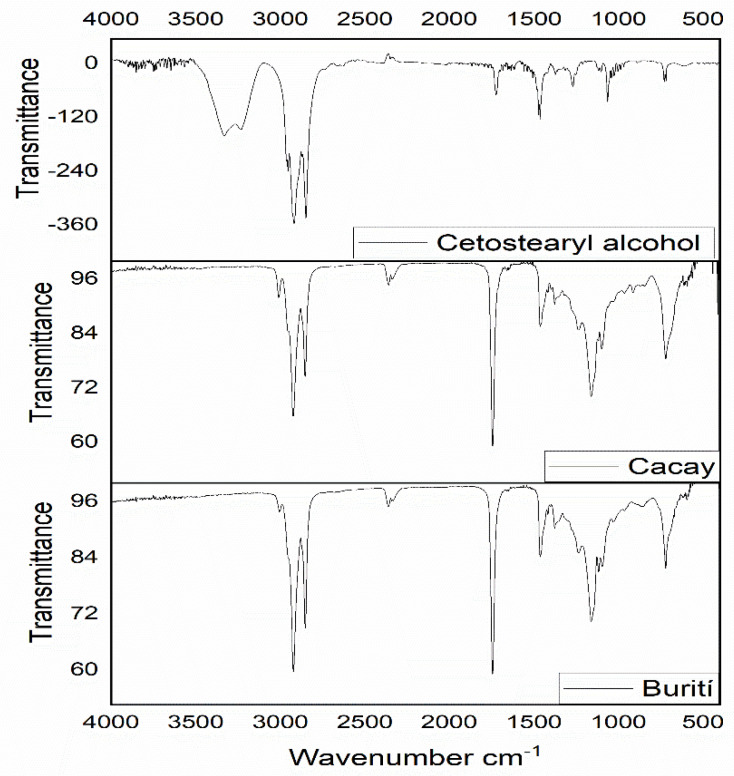
FTIR spectra of cetosteryl alcohol and Amazonian vegetable oils.

The understanding of the molecular interactions that occur in the organogels derived from the mixture of OA and CA can be analyzed with FTIR spectroscopy. The tests were performed on the organogel precursors and organogel samples synthesized with different OGG fractions. The spectra were used to investigate the intermolecular solvent/OGG or OGG/OGG interactions occurring during gelation. Weak solvent/OGG interaction results in dominant OGG/OGG interactions that can lead to the formation of a continuous network. However, a much stronger OGG/OGG interaction will eventually lead to the precipitation of clusters of crystalline molecules. Figure 5 shows the FTIR spectra of the pure oils. The most prominent peaks are C-H (asymmetric) stretching at 2922 cm^−1^, C-H (symmetric) stretching at 2852 cm^−1^, C-H scissors stretching at 1462 cm^−1^, C=O stretching peak at 1743 cm^−1^, C = C peak stretching at 1655 cm^−1^, = C-H peak stretching at 3006 cm^−1^, -CH_3_ and -CH_2_ peak stretching at 2852 cm^−1^, and -CH2 equilibrium peak stretching at 722 cm^−1^. Cetostearyl alcohol is a long-chain fatty alcohol mixture characterized by a single hydroxyl group. The FTIR spectrum for pure cetostearyl alcohol (Figure 5) showed some typical peaks observed for fats: -CH_2_ aliphatic group stretching vibrations between 2954 and 2918 cm ^−1^, -CH_2_ and -CH_3_ aliphatic group bending vibrations between 1463 and 1471 cm^−1^, -CH_2_ group bending vibrations at 1373 cm^−1^, and -CH_2_ group bending vibrations at 722 cm^−1^, while peaks at 3232 cm^−1^ and 3331 cm^−1^ showed the OH stretching mode.

**Figure 6:**
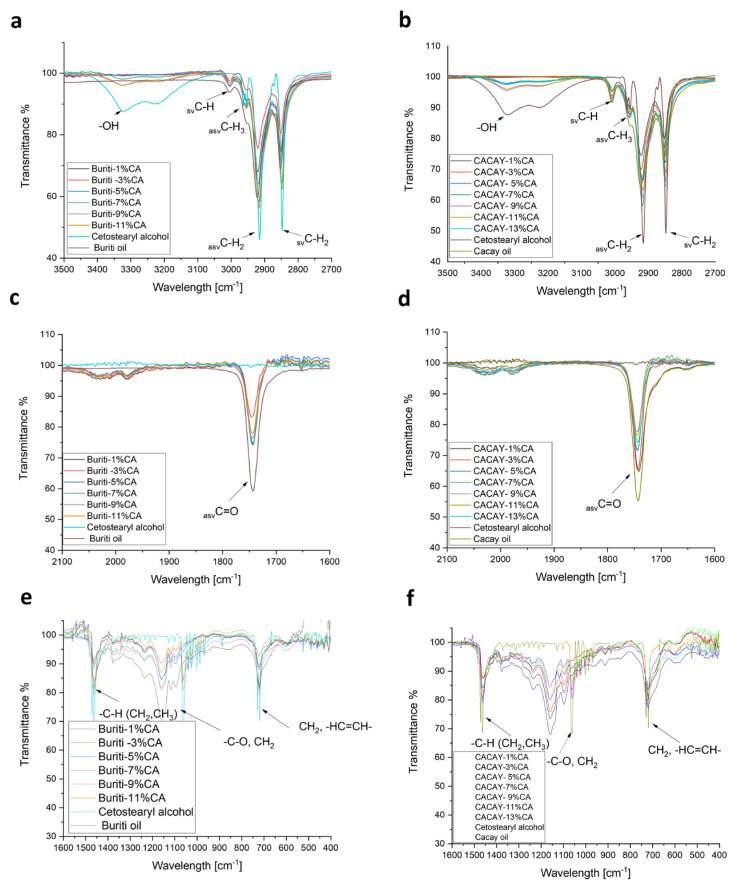
FTIR for each organogel and cetostearyl alcohol, (**a**,**b**) region between 3100–2800 cm^−1^; (**c**,**d**) region between 1800–1600 cm^−1^; and (**e**,**f**) region between 1500–600 cm^−1^.

The shape of the peaks appearing in the FTIR spectra of the organogels (Figure 6) is quite like those already described for pure oil. Three characteristic regions can be distinguished in the IR spectra for both the BO–CA organogel and the CO–CA organogel. The first region includes vibrations of non-polar aliphatic groups (3500–2700 cm^−1^) for gelation: it was observed that this gelator is mainly able to create van der Waals interactions, as evidenced by the shift in the absorption bands of symmetric and antisymmetric CH_2_ stretching vibrational modes, together with CH_3_ and CH (Figure 6 a,b). It is also observed that the asymmetric stretching vibrational absorption band of the CH_2_ group of the cetostearyl alcohol is more intense than this absorption band of the two organogels (BO–CA and CO–CA), indicating a longer hydrocarbon chain length of the alcohol. The most representative peak of cetostearyl alcohol is the OH between 3200 and 3400 cm^−1^, which is not observed at low concentrations and emerges gradually as the concentration of the organogelator increases. This is because the OH group acts as the origin of the heterogeneous nucleation, where the polar molecules of the Amazonian oils are associated by hydrogen bonds and van der Waals forces, generating a steric hindrance that hinders its visualization at lower concentrations. For the BO–CA and CO–CA organogels, the OH band is observed at 7% and 5%, respectively, indicating a saturation of the medium, increasing the solute–solvent interaction in the formation of the crystalline form. The second region includes the stretching absorption of the carbonyl group (2100–1600 cm^−1^); the C=O stretching peak appears at 1743 cm^−1^ for buriti oil, cacay oil, and organogels. Alcohol is characterized by no peak in the carbonyl group stretching absorption region, indicating that the position of this peak is mainly related to changes in the characteristics of the carbonyl groups in triglycerides; the intensity of this peak when the oil is pure is higher and when mixed with the organogelator the intensity is lower, indicating interactions to a greater or lesser extent when forming the 3D structure showing that this functional group is key in the formation of crystals and possibly in their size according to their nature.

The third region refers to the CH_2_ interactions that change their intensity, which may be mainly due to the hydrophobic interactions the samples undergo (Figure 6 e,f). Also, at 1160 cm^−1^, there is a variation in intensity from one oil to another; on the one hand, BO presents greater intensity in this region since it has more saturated compounds, that is, linear compounds that favor interactions; while CO, having more polyunsaturated compounds in cis, hinders such interactions. This behavior is also related to its crystallography since BO presents smaller crystals with greater surface area, indicating greater intensity, while CO presents larger crystals with low surface area and lower intensity.

### 3.6. X-ray Diffraction

X-ray diffraction was used to investigate the arrangement of the sheets of lipid molecules and the crystallinity of the fatty acid chains in the organogels; furthermore, XRD showed the presence of long-spaced peaks [001] and short-spaced peaks [hk0] [44]. The long and short spacing peaks provide information on the order and lateral packing of the molecular layers, respectively [45]. TAG fat crystals are classified into three polymorphic forms: α, β’, and β. Under supercooling conditions, a molten lipid begins to crystallize in the α-form, the least stable polymorph with the lowest melting point, rapidly transforming into the β’ state. The β’ form has the highest melting point and is the most stable crystalline state [44,45]. The peak positions and spacings of the diffractograms of the organogels are shown in Table 3.

**Figure 7:**
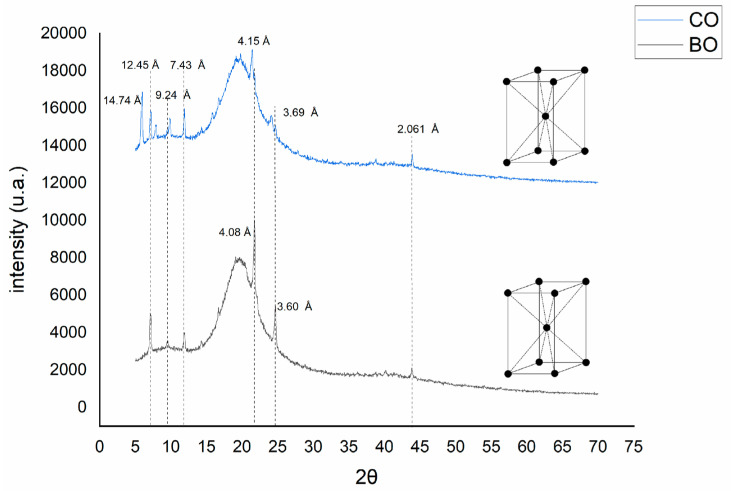
X-ray diffraction (XRD) of BO and CO organogels (CA 11%).

It was evident that there are three common peaks in the wide-angle region at around 2.06 Å, 3.60 Å, and approximately at 4.11 Å; these diffraction peaks are very similar to those reported in previous work by Dassanayake et al. [45] and Öğütcü et al., 2015 [35] where they work with waxes having significant amounts of long chain fatty alcohols [46]. From these results BO–CA and CO–CA organogels have similar behavior to TAG forming β’ polymorph with values of approximately 3.61 Å and 4.08 Å for BO–CA and 3.60 and 4.15 for CO–CA (Figure 7). The presence of palmitic acid (C16:0) in the system may have favored the conformation of the β’ phase in BO–CA organogels since this fatty acid is a promoter of this polymorphic phase [47]. The multiple peaks in the CO–CA organogel may be mainly due to its polymorphic conformation.

In the process of OG formation, it is possible for vegetable oils to be integrated into the OG molecule layers. It has been observed that the association of vegetable oils within CA layers affects the OG crystal size. In this context, as a function of peak width, the average crystal size (D) was calculated using the Debye–Scherrer Equation (2).

(2) $$D=\frac{K*\lambda }{\beta *\cos\theta }$$

where *K* is the Scherrer constant or shape factor, 0.89 (since the crystal shape is unknown); *λ* is the CuKα wavelength, i.e., 1.54 Å, *β* is the full width at half maximum (FWHM) of the diffraction peak in radians, and *θ* is the Braggs diffraction angle. The estimation and comparison of the average crystal size were performed using the peak at approximately 43.8 °2θ [110]. Adding oil showed a clear variation in CA crystal sizes (Table 3). Comparatively, the crystal sizes of BO–CA organogels were smaller than those of CO–CA organogels, which shows a correlation between the analysis performed and fractal dimensions in polarized light microscopy. Changes in crystal size of the CA crystals in the organogels confirms that vegetable oils are incorporated within the OGG layers.

### 3.7. Crystallization Evolution

**Figure 8:**
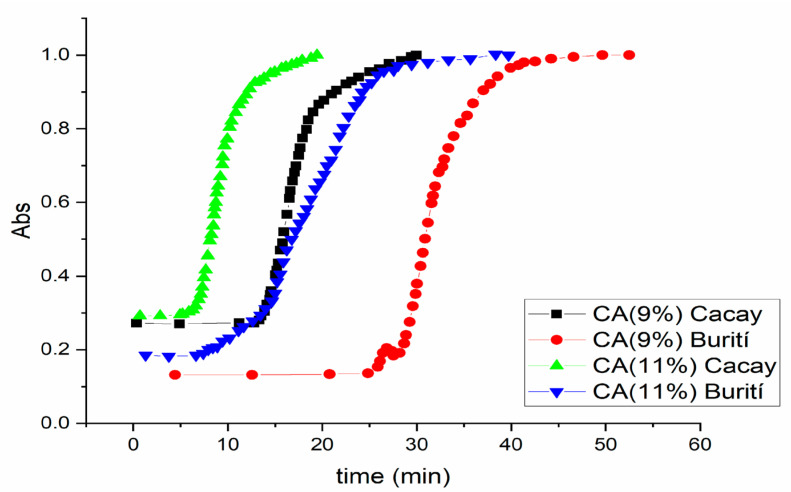
Evolution of gelation of CA–BO and CA–CO organogels.

Crystallization evolution (CE) is represented as the evolution of crystallization depending on time, so the gelification time is thought of as the time required to reach the equilibrium phase, where the absorbance of the gels does not increase with time. There are three phases in the crystallization kinetics: (i) the induction time (t1), which is generally influenced by increasing OGG concentration by the creation of more heterogeneous crystallization nuclei, reducing that induction time; (ii) the crystal growth time (t2), which is influenced by the molecular composition of the oils (commonly saturated fatty acids have a higher melting point than unsaturated ones) [36,42,45]; and (iii) the equilibrium time (t3), at which the end of crystallization is reached (Figure 8). The assays were first adjusted to the highest OGG concentrations where the organogel was formed (9% and 11%); in both cases, the shape of the adsorption profile was sigmoidal.

CA concentration influences by providing heterogeneous nucleation sites, stabilizes the developing nuclei, and affects the driving force [48]. This event occurs in BO and CO at 11% OGG concentration, faster than at 9%. The slow crystal growth time was approximately 6.58 min at 11% OGG for CO–CA organogel, which presented a lower amount of saturated fatty acids with lower melting points, and 18.4 min at 11% CA for BO–CA organogels, with a higher amount of saturated fatty acids and melting point (Table 4). The sigmoid profile and the long-term steady-state mean that the crystalline aggregates’ growth was one-dimensional and appears to have followed first-order kinetics. The one-dimensional growth of the aggregates results in the formation of long crystals with a high length–width ratio. Polarized light microscopy studies confirmed the formation of such crystals (Figure 2).

### 3.8. Thermal Analysis

**Figure 9:**
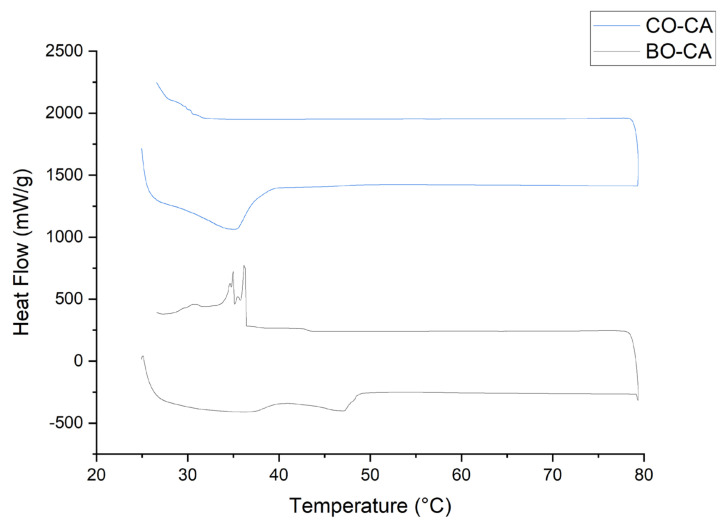
DSC plot for BO–CA and CO–CA organogels.

The melting and crystallization behavior of the organogel samples were tested up to 80 °C, where wide temperature ranges were observed (Figure 9). The occurrence of phase change over a wide temperature range suggested that the sol-to-gel and gel-to-sol transitions were not immediate but gradual. In Figure 9, we found the melting endothermy in the organogels occurs in two stages; the first presents the melting of the CA crystals that formed the gelling network, while the second one is its dissolution, which is related to the type of oil used. For CO, these stages are faster, presenting a single peak at about 36 °C, allowing the use of the formulations developed for topical applications. In the case of BO, these stages exhibited slower progression, accompanied by a broad endothermic peak.

Melting onset temperature (Tsm), melting temperature (Tm), crystallization onset temperature (Tsc), crystallization temperature (Tc), enthalpy of fusion (ΔHm), and enthalpy of crystallization (ΔHc) of organogels were calculated (Table 5). Enthalpy changes during the thermal events were measured by integrating the area under the curve. It was found that the onset temperatures and the change in enthalpies associated with the OG vary according to the nature and components of the oils; when these contained a higher number of saturated compounds associated with minor polar compounds, they presented a higher temperature, as is the case of BO–CA compared to CO–CA.

Microscopic observations suggested that the crystallization of the organogels was a nucleation and crystal growth process. Crystallization at the nucleation site was driven by non-covalent interactions between the CA molecules and the TAGs molecules of the vegetable oil, and this allowed the system to reach a low-energy state, releasing heat (an exothermic reaction). This driving force was counteracted by the entropic forces associated with the phase unmixing of the gelling crystals of the homogeneous solution. The change in entropies during the crystallization events was calculated using the Gibbs Equation (3):

∆G = ∆Hc − Tc × ∆Sc(3)

where ΔG and ΔS are Gibb’s free energy and the entropy change involved during crystallization, respectively. The change in entropies was calculated at the crystallization temperature, where ΔG tends to approach zero (Table 5) [36,49].

### 3.9. Rheological Analysis

**Figure 10:**
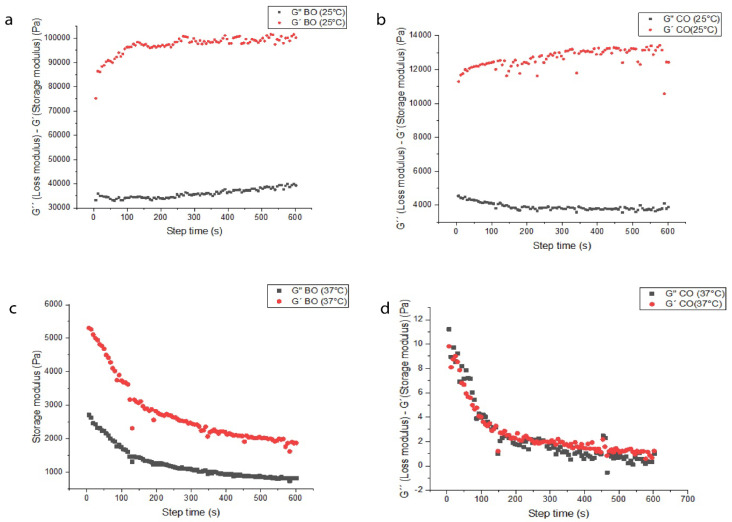
Mechanical spectrum of Amazonian oil organogels at a concentration of 11% OGG. (**a**) BO–CA organogel at 25 °C. (**b**) CO–CA organogel at 25 °C. (**c**) BO–CA organogel at 37 °C. (**d**) CO–CA organogel at 37 °C.

It was confirmed that the concentration of CA in the organogels influences directly on the self-assembly behavior of each of the oils. The BO–CA 11% formed self-assembled gels of higher consistency than the CO–CA 11% at a constant temperature of 25 °C (Figure 10). The increase in organogel rigidity is due to the concentration of OGG associated with the composition of the implemented AO. The value of storage modulus (G′) was higher than the value of loss modulus (G″), indicating the behavior of a semi-solid at 25 °C for both cases. Nevertheless, the values of G′ and G″ for the BO–CA organogel are significantly higher than for the CO–CA organogel, confirming the observed OBC data, in which the higher the amount of OGG, the better the gel consistency and less amount of OGG is required when the oil has a greater composition of saturated fatty acids.

**Figure 11:**
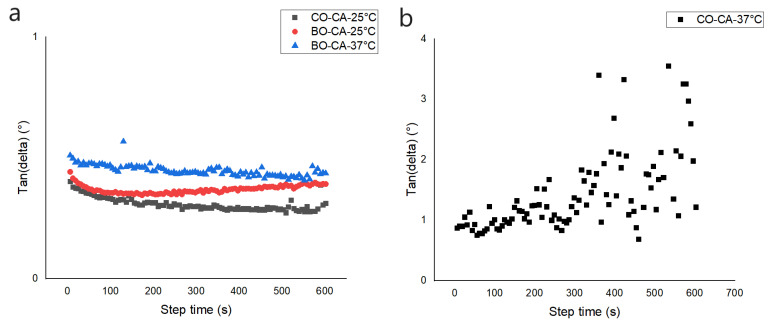
Tan δ as a function of time for organogels. (**a**) BO–CA (25 °C and 37 °C) and CO–CA (25 °C) organogels at 11% CA concentration. (**b**) CO–CA (37 °C) organogels at 11% CA concentration.

The tangent values of the phase angle (tan δ = G″/G′) validate the viscoelastic behavior of the organogels. This dimensionless measure compares the energy lost during the oscillatory test with the amount stored during this period and indicates whether the elastic or viscous property predominates. The tan(δ) values were closer for BO–CA organogels at 25 °C, BO–CA at 37 °C, and CO–CA at 25 °C. Related to gel behavior, it is of special interest that the behavior of BO–CA organogel at 37 °C is similar to that of CO–CA organogel at 25 °C. One plausible cause for this could be because a part of the BO–CA organogel crystals remains dissolved at this temperature, generating some free spaces that are natural in CO–CA organogel due to its initial composition. The CO–CA organogel at 37 °C tends to unite in its initial stage, so it is not considered a gel, and that is why its values oscillate (Figure 11).

## 4. Discussion

Organogels are systems that have a significant importance in the pharmaceutical field for the controlled release of drugs, improvement of bioavailability, and compatibility with biomaterials, among others, and their characterization is important to ensure the optimization of the formulation, its stability and the efficacy and safety of the finished product. Within the organogels prepared, it was observed that increasing the concentration of the organogelator (OGG) affects the physical properties, such as gel consistency and texture. The difference in CGC for each of the oils is due to the initial nucleation induced by the gelling agent and solvent molecules (TAGs). The TAGs molecules of BO have a high fraction of oleic acid (69.78%) and saturated fatty acids such as palmitic (21.96%) and stearic (5.03%). In contrast, CO contains a high fraction of polyunsaturated fatty acids, linoleic acid (75.1%) and monounsaturated fatty acids, oleic acid (11.8%), and in low concentration palmitic (9.5%) and stearic (2.2%) (Table 1). These differences lead to a change in the gelling capacity of the oils [50]. They may also affect the nucleation mechanism, and the consistency of the organogels formed [12].

The concentration of the gelling agent or organogelator plays a fundamental role by promoting the interactions between the TAGs to be more frequent as its concentration increases, requiring more quantity for the gelation of CO since it has fewer saturated compounds that serve as hydrophobic bridges in the nucleation. At the same time, for BO, there are more saturated compounds inducing more interactions, and consequently, the formation of the gel is presented with a lower concentration of CA [51,52]. Some authors explain this change because low OGG concentrations induce nucleation in long-chain saturated molecules (between 16 and 18 carbon atoms), due to hydrophobic interactions and Vander Wall forces. As the concentration increases, it induces nucleation in other fatty acids, generating greater solvent–OGG interaction, and on the same OGG–OGG, making crystals more structured and consequently larger [53].

Subsequently, a microscopic to macroscopic approach was carried out to determine the unique molecular nature of each Amazonian oil to understand how the amount of saturated and unsaturated fatty acids, and minor polar components, among others, can affect the microstructural properties such as size, shape, and mass distribution of the organogels. Fractal dimension (DB) analysis was performed using the box-counting method, which allows us to numerically estimate the uniformity of the solid mass distribution in the crystal lattice of the organogel. For this analysis, the main criteria considered was the concentration close to the formation of the organogel, which enabled us to accurately visualize the crystals within the organogel. The identified concentration was 9% for both BO–CA and CO–CA organogels.

The distribution and size of the crystal that forms within the 3D structure are two factors that affect consistency, which is given by the oil-binding capacity of the OGG. This statement is confirmed with the value of the lacunarity (A) and crystal length (Lc) for the CO–CA organogel, which suggests a more irregular structure since TAGs are constituted by mixtures of mostly polyunsaturated and monosaturated fatty acids, generating large crystals with many empty cavities, thus leaving more space between the structures showing low oil binding capacity, leading to the low strength of the 3D structure that with shear stresses could be easily fragmented. A large number of empty cavities is usually related to larger crystals, while smaller crystals with a larger surface area present a much more stable structural organization because they are better distributed in the 3D structure [43]. It has already been established that the most stable polymorphic form of triacylglycerol POP (1,3-dipalmitoyl -2- oleyl glycerol), which is a mixture of saturated and unsaturated fatty acids, is a lamella since the chains of palmitic and oleic fatty acids are laid in different lamellar planes. It was confirmed for the two oils that the crystal distribution and size are related to the OGG concentration; at lower OGG concentrations, the oils did not form a stable structure. Therefore, this leads to low oil binding capacity, leading to phase separation. Furthermore, when the OGG concentration value is high, the ability of the organogels to form 3D structures is better. We ensure this after centrifugation, where it reflects high stability and, consequently, higher oil binding capacity.

The time required for organogel setting is another factor that is affected by the oil constituents. The slow rate of initial transformation can be attributed to the time required to form a significant number of crystallization nuclei and for them to have the appropriate size for diffusion. The variation in gelation time between BO and CO could be associated with differences in composition in terms of TAGs and minor polar compounds. The first crystallization stage often involves fully saturated TAGs, while the second stage is usually the crystallization of partially unsaturated TAGs (commonly monounsaturated). Cores composed mainly of highly saturated TAGs do not necessarily provide a good growth base for partially unsaturated fat [48]. The concentration of polar minority compounds can affect the first stage of crystallization, especially in CO, which has a higher amount of free fatty acids than BO.

Entropy changes develop due to crystal–solvent interfacial tension and form a key parameter for core formation during crystallization [48,54], coupled with OGG oversaturation, thus generating heterogeneous nucleation [55]. The entropy loss was higher in the BO–CA associated organogel with a higher long-chain saturated fatty acid content. Therefore, this oil leads to the formation of organogels with higher thermodynamic stability. The exothermic peaks observed in the BO exotherm correspond to differences in the crystallization temperature of the oil constituents. The increase in entropy loss indicates a reduction in the crystal–solvent interfacial tension. In other terms, the crystal–solvent interfacial tension decreases when the oil has high concentrations of saturated fatty acids. As can be observed, for both BO–CA organogel and CO–CA organogel, several crystallization peaks corresponding to α, β, and β’ crystalline forms were found due to their high cooling rate (10 °C/min). However, peaks were more intense for the BO–CA organogelator because of its higher enthalpy change suggesting that the kinetic strength of the system decreased rapidly giving no time for the molecules to align properly and form the unstable polymorphic form [5441].

As mentioned above, the hydroxyl group of cetostearyl alcohol interacts through intermolecular hydrogen bonds, which minimizes interfacial tension by hiding polar groups and exposing hydrophobic tails to vegetable oils. Such minimal interfacial interactions lead to a decrease in surface tension, which is the thermodynamic driving force for the formation of stable nuclei. This orientation results in the bilayer arrangement of the cetostearyl alcohol molecules during crystal formation.

In rheological analysis, the differences in the response of G′ for the organogels indicate higher or lower molecular structure and consequently higher or lower molecular weight, in addition to the increase or decrease in stiffness due to the alteration of the molecular structure. The BO–CA organogel’s G′value indicates a lower molecular structure with higher solid content and stiffness. Meanwhile, the CO–CA organogel shows a higher molecular structure with lower solid content, confirming what was observed in the fractal dimension analyses. When shear stress is exerted on larger crystals, it generates fragmentation into smaller crystals with large oil spaces. Variations and concentrations in the composition of fatty acids and polar minority compounds in the AO resulted in different crystallization mechanisms and, therefore, in differences in the strength of the organogels.

At a temperature of 37 °C (body temperature), the analysis of the organogels revealed changes in the values of G′ and G″. The BO–CA organogel presented a drop in both values, maintaining its behavior as a gel for a certain time. In contrast, the CO–CA organogel showed substantially lower and very similar G′ and G″ values, losing its semi-solid behavior, evidencing the loss of structural integrity as a function of temperature. The BO–CA organogel contains high melting point fatty acids, generating a higher structural resistance at elevated temperatures.

Due to the sensitivity of the Amazonian oils, it is recommended to perform a stability analysis of the organogels with IR, since during this analysis a variation of the peaks in the region of the carbonyl groups was observed, possibly due to a degradation caused by the cooling and heating cycles throughout the tests carried out [56].

In terms of the potential applications of organogels, the use of Amazonian oils in the cosmetic industry has shown promise. These oils can be employed as a moisturizer, offering a luxurious texture and enhanced skin absorption compared to traditional vegetal oil which generally are refined oils. This makes them suitable for both face and body applications. Organogel made from Amazonian oils can also stabilize active ingredients, improving their penetration into the skin [4]. This is particularly advantageous for serums containing antioxidants, vitamins, and peptides, as some of these beneficial components are naturally found in Amazonian oils [12]. Additionally, the use of Amazonian oil organogel in eye creams provide a smooth and lightweight texture that is easy to apply around the delicate eye area [57]. Lastly, organogels can enhance the dispersion and stability of UV filters, resulting in more even and effective coverage compared to traditional emulsions [58,59].

## 5. Conclusions

The composition of Amazonian oils, mainly triacylglycerols of saturated and unsaturated fatty acids, followed by minor polar compounds and unsaponifiable compounds, makes them complex organic solvents that directly influences both the crystallization processes and the nature of the structuring material. It was found that a higher amount of saturated chain triacylglycerols results in a lower amount of organogelator that is required, giving rise to small lamellar-shaped crystals and a high melting point. Additionally, the presence of monounsaturated and polyunsaturated triacylglycerols requires a higher amount of organogelator, generating polymorphic crystals of larger size with more empty spaces between them, resulting in lower melting points and reduced physical stability. Cetostearyl alcohol generates heterogeneous crystallization nuclei due to the presence of a polar group (OH-), causing triacylglycerols to overlap due to Van Der Walls and hydrophobic forces, overcoming the forces necessary for crystal generation. The crystal growth is a function of the amount of organogelator and free fatty acids, which promotes the existence of more crystallization nuclei. The physical and thermal stability of the organogel depends on the 3D structure formed; thus, the size of the crystals, their shape, and the space between them result in the final characteristics for its application. The use of Amazonian buriti oil to manufacture organogels produces crystals of smaller lamellar size, with fewer empty spaces, providing physical and thermal stability due to the balance between saturated and unsaturated fatty acids appropriate for dermal application. The organogel made from cacay oil has larger crystals with many empty spaces, which makes it highly physically unstable; moreover, its polyunsaturated fatty acids contribute to a low melting point, resulting in diminished thermal stability. Amazonian oils as a solvent in the structuring of organogels result in microstructures with physical properties different from those obtained with other organic solvents. The novelty and significance of developing organogels with Amazonian oils, in addition to generating a stable formulation, includes increasing the number of beneficial properties for the skin due to the nutrients present in the oils, being an ideal vehicle for bioactive substances which are protected by the 3D structure. The gel acts as a three-dimensional matrix that envelops the bioactive substance, separating it from the environment. This can isolate the bioactive substance from degradation agents such as oxygen, moisture, and light, reducing its exposure to these factors and thus creating a physical and diffusion protection barrier. This structure is also favored by the polar minority components and unsaponifiable compounds of the unrefined Amazonian oils [12,60], opening the doors to an important area of research, mainly driven by the possible use of these functional organogels as photo protectors of compounds that function as key bioactive substances (Vitamin A, Vitamin E and antioxidant compounds). Also, they could be a possible vehicle to transport pharmaceutical or dermo-cosmetic active ingredients, using the controlled release mechanism, which is based on the gel’s ability to regulate the diffusion of the active substance (bioactive) from its three-dimensional matrix into the surrounding environment. Organogels can adjust the rate of release of the bioactive substance by controlled diffusion, degradation of the organogel, and temperature change, among others [57,61].

## Figures and Tables

**Table 1:** Fatty acid profiles and quality indices of buriti and cacay oil.

Fatty Acid	Cacay Oil	Buriti Oil
Palmitic (C16:0) (%)	9.5	21.96
Stearic (C18:0) (%)	2.2	5.03
Oleic (C18:1 *cis*9) (%)	11.8	69.78
Linoleic (C18:2 *cis*9,12) (%)	75.1	ND
Unsaturated fatty acid (UFA)	86,9	69.78
Saturated Fatty acid (SFA)	11.7	26.99
Monounsaturated fatty acid (MUFA)	11.8	69.78
Free acidity (%)	4.583 ± 0.09	2.653 ± 0.05
Saponification index (mgKOH/g)	188.862 ± 8.605	190.405 +/− 2.412
Refractive index (25 °C)	1.464 ± 0.002	1.4629 ± 0.00005
Viscosity (Cp)	137.4 ± 0.00308	180.76 ± 0.0026
Relative density (kg/m^3^)	0.908 ± 0.001	0.905 ± 0.0004

ND = Not detected.

**Table 2:** Crystal length (Lc), fractal dimension (DB), and lacunarity (A) values for 9% OGG in BO–CA and CO–CA organogels via the box-counting method.

Sample	Image	Crystal Length Lc (µm)	Dimension Fractal (D_B_)	Lacunarity (A)
BO–CA-9%	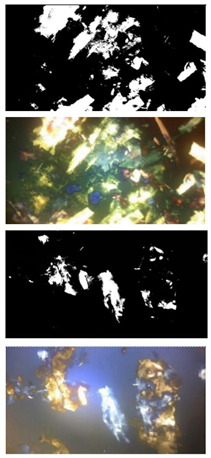	169.05 ± 1.7263	1.595 ± 0.0184	1.0116± 0.045
CO–CA-9%		226.20 ± 1.577	1.379 ± 0.0110	2.046± 0.026

**Table 3:** XRD data of CO and BO organogel samples were divided into long-spacing peaks and short-spacing peaks.

Sample	Type of Peaks	Peak Position (°2θ)	d-Spacing (Å)	FWHM of Peak (°2θ)	D (nm)
CO	Long spacing peaks	5.99	14.74	0.23	59.48
		7.15	12.36	0.21	69.43
		7.88	11.21	0.24	56.94
		9.56	9.24	334.07	0.02
		9.85	8.97	0.49	20.30
		11.90	7.43	0.20	80.59
	Short spacing peaks	21.39	4.15	0.21	71.77
		24.10	3.69	0.34	33.09
		24.68	3.60	0.19	90.40
		43.89	2.06	0.16	134.81
BO	Long spacing peaks	7.09	12.45	0.26	48.04
		9.56	9.24	0.47	21.41
		11.90	7.43	0.22	65.80
	Short spacing peaks	21.75	4.08	0.21	73.90
		24.65	3.61	0.25	54.48
		43.86	2.06	0.22	67.72

**Table 4:** Organogel gelation parameters include induction time (t1), crystal growth time (t2), and equilibrium time (t3).

Oil	Concentration of the Gelator	Δt1(min)	Δt2 (min)	Δt3 (min)
**Cacay oil**	9%	13.53	6.11	10.36
	11%	6.28	6.58	5.84
**Burití oil**	9%	26.42	13.48	15.1
	11%	7.38	18.47	15.17

**Table 5:** Thermal properties of organogels obtained with DSC.

Sample	Melting, Endotherm				Crystallization, Exotherm			
	Tsm (°C)	Tfm (°C)	ΔHm (J/g)	ΔSm (J/g)	Tsc (°C)	Tfc (°C)	ΔHc (J/g)	ΔSc (J/g)
Burití	41.90	47.12	5.8101	0.0349	36.47	29.26	7.3085	0.2497
Cacay	26.63	35.23	8.6196	0.244	30.68	27.66	0.2221	0.0073

## Data Availability

All data relating to the present study can be requested from the author for correspondence.
